# Supplementary material for: Correction of Breech Presentation with Moxibustion and Acupuncture: A Systematic Review and Meta-Analysis
Source: Healthcare (Basel). 2021 May 22;9(6):619. doi: 10.3390/healthcare9060619 (PMC8224784; doi:10.3390/healthcare9060619)

Supplementary Materials:

Table S1. Literature search strategy

Database	Search strategy	
PubMed	1.	breech
	2.	breech presentation [Mesh]
	3.	labor Presentation [Mesh]
	4.	non-cephalic OR noncephalic
	5.	non-vertex OR nonvertex
	6.	acupuncture [Mesh]
	7.	acupuncture therapy [Mesh]
	8.	Electroacupuncture OR Electro-acupuncture
	9.	acupressure
	10.	moxibustion [Mesh]
	11.	artemisia vulgaris
	12.	mugwort
	13.	Version, Fetal [Mesh]
	14.	1 OR 2 OR 3 OR 4 OR 5
	15.	6 OR 7 OR 8 OR 9 OR 10 OR 11 OR 12
	16.	14 AND 15
	17.	13 AND 14 AND 15
MEDLINE	1.	breech.mp
	2.	breech presentation/
	3.	labor Presentation/
	4.	non-cephalic OR noncephalic
	5.	non-vertex OR nonvertex
	6.	acupuncture/
	7.	acupuncture therapy/
	8.	Electroacupuncture OR Electro-acupuncture
	9.	acupressure.mp
	10.	moxibustion/
	11.	artemisia vulgaris.mp
	12.	mugwort.mp
	13.	Version, Fetal/
	14.	1 OR 2 OR 3 OR 4 OR 5
	15.	6 OR 7 OR 8 OR 9 OR 10 OR 11 OR 12
	16.	14 AND 15
	17.	13 AND 14 AND 15
Embase	1.	breech/exp
	2.	breech presentation
	3.	labor presentation
	4.	non-cephalic OR noncephalic
	5.	non-vertex OR nonvertex
	6.	acupuncture/exp
	7.	electro-acupuncture OR electroacupuncture
	8.	acupressure
	9.	moxibustion/exp
	10.	artemisia vulgaris
	11.	mugwort
	12.	fetal version/exp
	13.	1 OR 2 OR 3 OR 4 OR 5
	14.	6 OR 7 OR 8 OR 9 OR 10 OR 11
	15.	13 AND 14
	16.	12 AND 13 AND 14

Cochrane Library	1.	breech
	2.	MeSH descriptor: [Breech Presentation] explode all trees
	3.	MeSH descriptor: [Labor Presentation] explode all trees
	4.	non-cephalic OR noncephalic
	5.	non-vertex OR nonvertex
	6.	MeSH descriptor: [Acupuncture] explode all trees
	7.	MeSH descriptor: [Acupuncture Therapy] explode all trees
	8.	Electroacupuncture OR Electro-acupuncture
	9.	acupressure
	10.	MeSH descriptor: [Moxibustion] explode all trees
	11.	artemisia vulgaris
	12.	mugwort
	13.	MeSH descriptor: [Version, Fetal] explode all trees
	14.	1 OR 2 OR 3 OR 4 OR 5
	15.	6 OR 7 OR 8 OR 9 OR 10 OR 11 OR 12
	16.	14 AND 15
	17.	13 AN D 14 AND 15

Table S2. Summary of risk of bias assessment

Domains	Descriptions
Allocation	All included studied mentioned randomization. Six studies reported method of randomization and allocation concealment [10, 15, 16, 25, 26, 29]. Six studies did not report how patients were randomized or how the allocation sequence was concealed [11, 17, 19, 21, 24, 27]. One study had baseline imbalances that might indicate problems with randomization process [28]. Another study reported neither randomization process nor allocation concealment process [22]. Two studies did not state how the allocation sequence was concealed and the allocation sequence was not random due to patients were assigned by date of admission [20, 23].
Performance	The review authors considered that lacking of blinding may not affect the outcome, because objective outcome was addressed in these studies. Five studies managed to provide further information about co-interventions [15, 17, 25, 26, 28], and the remaining trials did not.
Follow-up	One study had a relatively high drop-out rate (>20%), which may influence the outcome [25].
Measurement	One trial did not state how fetal malposition was assessed [22], and the others reported how the outcome was measured. The review authors judged that whether outcome assessors were blind or not, the outcome are not likely to be influenced.
Reporting	Three trials reported study protocol [10, 15, 25], and the remaining trials failed to report protocols.

Figure S1. Forest plot of comparison: Moxibustion versus Control; Outcome: Preterm delivery & Premature rupture of membranes.

Preterm delivery:

Study or Subgroup	Treatment Events	Treatment Total	Control Events	Control Total	Weight	Risk Ratio M-H, Random, 95% CI
Cardini 2005	2	65	1	58	19.9%	1.78 [0.17, 19.17]
Do 2011	2	10	0	10	13.1%	5.00 [0.27, 92.62]
Vas 2013	4	136	5	134	67.0%	0.79 [0.22, 2.87]
**Total (95% CI)**		**211**		**202**	**100.0%**	**1.18 [0.41, 3.41]**
Total events	8		6			

Heterogeneity: Tau² = 0.00; Chi² = 1.45, df = 2 (P = 0.48); I² = 0%
Test for overall effect: Z = 0.31 (P = 0.76)

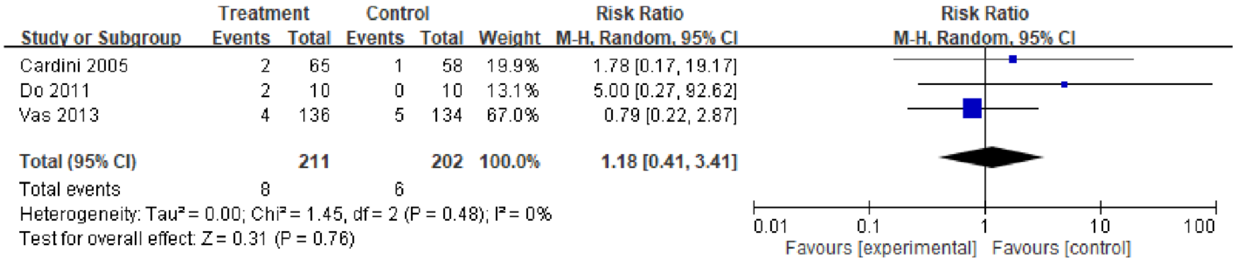

Premature rupture of membranes:

Study or Subgroup	Treatment Events	Treatment Total	Control Events	Control Total	Weight	Risk Ratio M-H, Random, 95% CI
Cardini 1998	10	130	8	130	84.2%	1.25 [0.51, 3.07]
Cardini 2005	2	65	0	58	7.5%	4.47 [0.22, 91.22]
Do 2011	3	10	0	10	8.4%	7.00 [0.41, 120.16]
**Total (95% CI)**		**205**		**198**	**100.0%**	**1.59 [0.70, 3.62]**
Total events	15		8			

Heterogeneity: Tau² = 0.00; Chi² = 1.83, df = 2 (P = 0.40); I² = 0%
Test for overall effect: Z = 1.10 (P = 0.27)

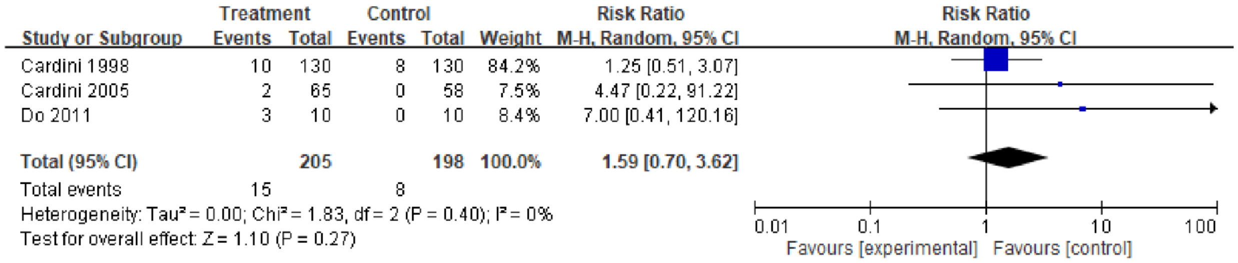

Supplement: Supplementary file 1 [file healthcare-09-00619-s001.zip › healthcare-1200287-supplementary.pdf]
